# Biomimetic hair-assisted GaN optical devices for bidirectional airflow detection

**DOI:** 10.1038/s41378-024-00794-2

**Published:** 2024-11-08

**Authors:** Tianyu Ye, Jian Chen, Xinke Tang, Kwai Hei Li

**Affiliations:** 1 School of Microelectronics, Southern University of Science and Technology, 518055 Shenzhen, China; 2 Pengcheng Laboratory, 518055 Shenzhen, China

**Keywords:** Optical sensors, Electrical and electronic engineering

## Abstract

Airflow sensing plays a pivotal role in numerous fields, including medicine, industry, and environmental monitoring. However, detecting bidirectional airflow using a single sensing unit poses significant challenges. In this work, a miniature airflow sensing device is introduced, utilizing a GaN optical chip integrated with a biomimetic hair structure. The sensing device comprises a monolithic GaN chip that handles both light emission and detection. The biomimetic hairs, constructed from nylon fibers and PDMS film, undergo structural bending in converting airflow signals into optical changes, modulating the light captured by the on-chip detector. The intensity of the airflow directly correlates with the bending extent of the biomimetic hair, facilitating the precise detection of airflow rates through changes in the photocurrent. The integrated device can measure a wide range of airflow rates from −23.87 ms^−1^ to 21.29 ms^−1^, and exhibit a rapid response time of 13 ms and a detection limit of 0.1 ms^−1^. Characterized by its compact size, fast response time, and bidirectional detection ability, the developed device holds immense potential for applications in breath detection, speech recognition, encoding information, and the realization of logic operations.

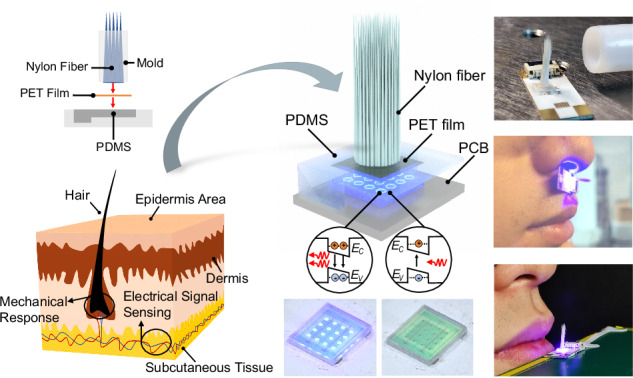

## Introduction

The detection of airflow rate holds great significance across diverse fields, spanning from urban settings and aircraft applications to human microenvironments and biomedical engineering^1–5^. In recent years, biomimetic hair sensors have emerged as promising tools for airflow detection, exhibiting various sensing mechanisms such as piezoresistive^6–10^, piezoelectric^11,12^, capacitive^13,14^, photoelectric^15^, and magnetic types^16,17^. Although advanced materials and structures such as electrospinning materials^18^, carbon nanotubes^19–21^, and graphene^22^ have been proposed for enhancing the detection ability, achieving a fast response, a wide measurement range, and bi-directional airflow detection remains a challenge. Optical fiber-based airflow sensors^23–26^ with wide measurement range, high sensitivity, and fast response time^24,27^ have attracted extensive research interest. Fiber-optic airflow sensors involve assembling external components that require precise optical alignment and other conditions. However, it may increase the complexity and size of the sensing system and limit practical applications.

GaN and its alloys are excellent candidates for fabricating light sources and detectors, attributed to their high efficiency, stability, longevity, and fast response time^28,29^. However, the inherent stiffness of GaN devices poses a significant hurdle in their ability to respond effectively to airflow. Currently, there are only a few reports on GaN-based airflow sensing devices, typically limited to detecting airflow in a single direction^30^. In this study, a promising bi-directional airflow sensing device is introduced through a GaN-based chip integrated with a biomimetic hair structure. The GaN chip acts as a core component for light emission and detection. Nylon fibers and PDMS are employed as biomimetic hairs. Nylon fibers are cost-effective, structurally flexible, and chemically stable. Among the various elastomeric materials, PDMS offers several advantages, including simplicity of handling, remarkable elasticity, ease of fabrication, and biocompatibility. Moreover, the PDMS can be directly and firmly integrated with the nylon fibers through a simple curing process, which reduces structural complexity and enhances the compactness of the sensing device. The hair structure responds to bidirectional airflow, bending to introduce optical signals captured by the integrated photodetector. A comprehensive investigation into the electrical and optical properties of the device, alongside its ability to detect different airflow rates, is conducted to validate the effectiveness of the proposed design.

## Results and discussion

**Fig. 1:**
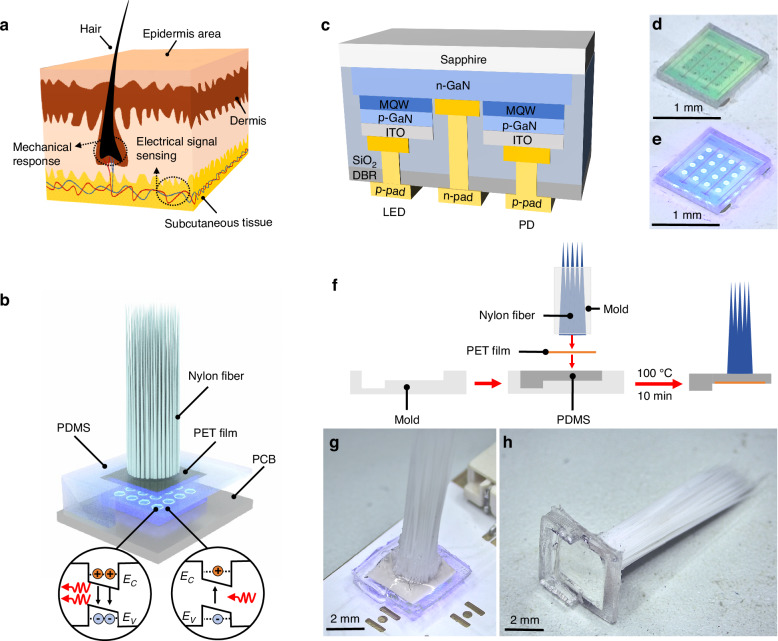
Structure of the biomimetic hair-assisted GaN optical devices. Schematic diagrams of **a** the skin structure and **b** the airflow sensing device. **c** Schematic view of the GaN chip. Microphotographs of the GaN chip in **d** non-luminous and **e** luminous states. **f** Schematic diagrams showing the integration of nylon fibers, a PDMS film, and a PET reflective film. Optical images of **g** the resultant sensing device and **h** the biomimetic hair structure

The biomimetic hair structure is designed to mimic the human hair skin shown in Fig. 1a for precise airflow detection. When human hair encounters airflow, it exhibits a mechanical response that the skin subsequently converts into electrical signals, enabling humans to perceive the presence and strength of the airflow. Figure 1b schematically shows the composition of the proposed sensing device, which consists of nylon fibers, PDMS film, PET reflective film, and a GaN chip. The nylon fibers and PDMS film serve as analogues for human hair and skin, respectively. The PET reflective film has a high reflectance of 95% and deformation recoverability. Figure 1c shows the schematic structure of the GaN chip, and its optical images in non-emitting and emitting states are shown in Fig. 1d, e, respectively. Figure 1f illustrates the process of fabricating the nylon fiber and PET reflective film integrated with PDMS film. The resulting biomimetic hair structure and integrated sensing device are presented in Fig 1g, h, respectively.

**Fig. 2:**
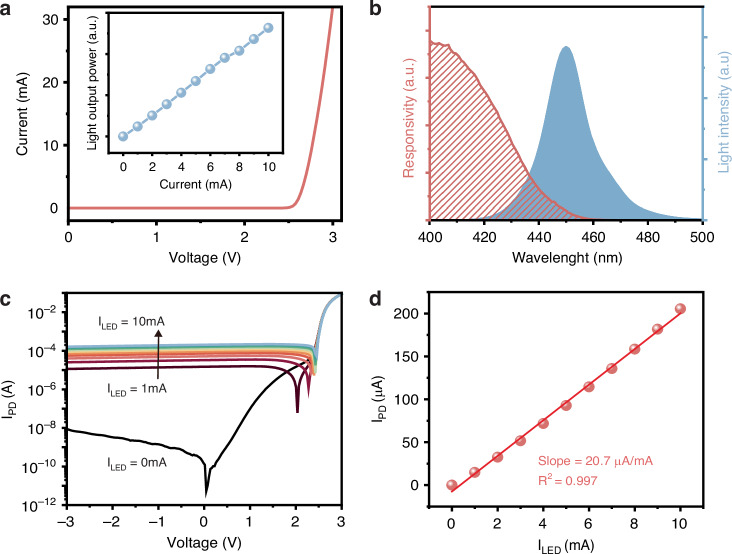
Optical and electrical properties of the GaN chip. **a** *I–V* characteristic curve of the LED. The inset shows the light output power as a function of the LED drive current. **b** Emission spectra of the LED biased at 10 mA and normalized spectral responsivity of unbiased PD. **c** *I–V* curve of PD measured at different LED currents. **d** Plot of the PD photocurrent as a function of the LED current. The solid line represents the linear fit to the data points

The electrical properties of the GaN chip are assessed using a sourcemeter (2450, Keithley), offering a current resolution of 50 pA. At a forward-biased current of 10 mA, the LED exhibited a voltage of 2.77 V, and the resistance, calculated from the inverse slope of the I-V curve, is found to be 11.49 Ω, as illustrated in Fig. 2a. The figure inset also reveals a linear correlation between the light output power of the LED and the injected current. Subsequently, optical inspection is performed to verify the luminescence capabilities of the LED and the responsivity of the PD. The emission spectrum of the LED, presented in Fig. 2b, features a peak wavelength of approximately 450.2 nm and a full width at half-maximum of about 17.7 nm. The photoresponse curve of the PD gradually declines with increasing wavelength, intersecting with the emission spectrum of the LED in the range of roughly 420 nm to 460 nm. This spectral overlap is attributed to the absorption edge shifts arising from the quantum-confined Stark effect and the band-tail effect caused by indium fluctuations within the InGaN/GaN MQWs^31^. In the absence of LED illumination, the dark current of the PD, measured under reverse bias conditions, is in the order of 10^−8 ^A, as shown in Fig. 2c. When a bias current ranging from 1–10 mA is applied to the LED, the photocurrent reaches an order of magnitude between 10^−5^ and 10^−4 ^A. Figure 2d shows a relationship between the PD photocurrent and the LED current. The solid line represents the linear fit to the data points, with an R^2^ value of 0.997, implying the responsiveness of the PD to the variations in LED emission intensity.

**Fig. 3:**
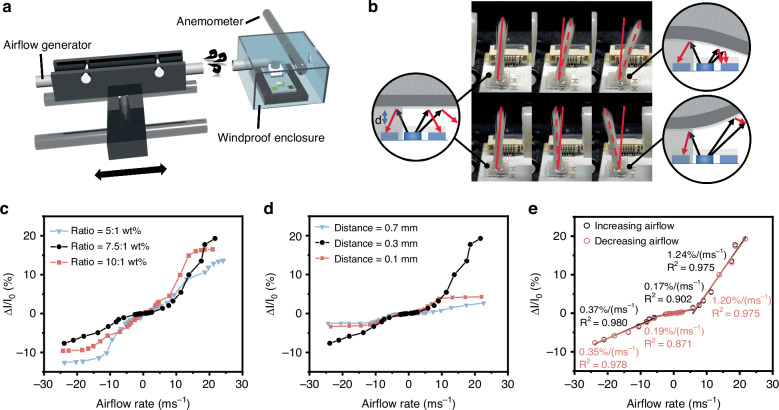
Working principle and characteristics of the airflow sensing device. **a** Schematic diagram showing the experimental setup for airflow measurement. **b** Optical images and schematic diagrams of the sensing device under positive and negative airflow. **c** Photocurrent as a function of airflow rate for the devices with different ratios of PDMS elastomer and curing agent. **d** Photocurrent as a function of airflow rate for the devices with different distances between the chip and reflective film. **e** Plot of the relationship between the photocurrent and airflow rate when *D* is 0.3 mm and the PDMS ratio is 7.5:1 wt%

The response of the sensing device to airflow is then evaluated. Figure 3a schematically illustrates the experimental configuration of airflow measurement. The air pipe connecting the airflow generator is mounted on a linear motorized stage. By adjusting the position of the outlet pipe, the airflow rate to the device can be precisely controlled. The airflow rate readings are calibrated using a hot-wire anemometer (425, Testo), capable of resolving airflow rates down to 0.01 ms^−1^. Although the sensing device is insensitive to changes in ambient humidity (see Supplementary Information S1), it is known that the optical and electrical properties of the GaN chip can be influenced by temperature variations^32^. Therefore, experimental measurements are conducted under controlled laboratory conditions, maintaining a constant temperature of 25 °C.

Schematic diagrams in Fig. 3b illustrate the distribution of reflected light within the device under varying airflow conditions. The light emitted from the LED reaches the PET reflective film through the transparent sapphire substrate. In the absence of airflow, the unbent nylon fibers maintain the reflective film in a nearly horizontal state, reflecting a moderate amount of light onto the PD. When the positive airflow exists, the nylon fibers bend and drive the reflective films closer to the chip, allowing the PD to receive an increased amount of light. Conversely, a negative airflow direction causes the nylon fibers to lift the reflective film upwards, resulting in diminished light reception by the PD. The polarity of the photocurrent signal from the PD serves as an indicator of airflow direction, while the photocurrent magnitude provides a quantitative representation of the airflow rate.

One factor that impacts the sensing performance is the stiffness of the PDMS layer beneath the biomimetic hair structure^33^, which affects the proximity between the PET film and the GaN chip when airflow is applied. The elastic modulus and stiffness of the PDMS layer can be readily adjusted by controlling the ratio of the PDMS elastomer matrix and curing agent. Figure 3c shows the relationship between airflow rate and photocurrent, which varies depending on the PDMS mixture ratio. When the airflow rate exceeds 15.87 ms^−1^, there is a limited change in photocurrent of about 0.1% for a ratio of 10:1 wt%. To extend the measurement range of the device, one effective strategy is to reduce the mixture ratio, thereby increasing the stiffness of the PDMS layer. At ratios of 7.5:1 wt% and 5:1 wt%, the measurement ranges expand to about −23.87 ms^−1^ to 21.29 ms^−1^ and −13.43 ms^−1^ to 24.43 ms^−1^, respectively. It is worth noting that the PDMS mixture with a ratio of 7.5:1 wt% achieves the highest elastic modulus, aligning with previous research findings reported in ref. ^34^. PDMS with higher stiffness requires a more substantial airflow to attain its maximum deformation, thereby extending the detectable airflow range. Therefore, the optimal PDMS mixture ratio is selected as 7.5:1 wt%. Although the measurement range can potentially be extended by further increasing the stiffness of the PDMS layer, there exist tradeoff in reducing the sensitivity and detection limit of the device.

The initial distance of the reflective film from the chip is another crucial factor that affects the intensity of the reflected light. Figure 3d shows the relationship between airflow rate and photocurrent at different initial distances (*D*). When a *D* value of 0.7 mm is established, a photocurrent variation of approximately 5.2% is observed within the airflow rate range of −24.35 ms^−1^ to 22.63 ms^−1^. Notably, the photocurrent remains nearly unchanged for airflow rates exceeding 13.21 ms^−1^ and falling below −11.78 ms^−1^. One potential approach to expand the measurement range is to reduce the *D* value by positioning the PET film closer to the chip surface. When *D* decreases to 0.3 mm, the measurement range can be enhanced to −23.87 ms^−1^ to 21.29 ms^−1^. However, further reducing *D* to 0.1 mm restricts the ability of the device to detect airflow, limiting it to a range of −10.21 ms^−1^ to 9.16 ms^−1^. Therefore, the PDMS ratio is chosen as 7.5:1 wt%, and the D is set to 0.3 mm to ensure the widest possible measurement range.

After optimizing the structural parameters of the integrated device, the relationships between airflow rate and the photocurrent under different airflows are measured. A linear fitting analysis is performed for three intervals, as shown in Fig. 3e. For the airflow rates ranging from −23.87 ms^−1^ to −5.89 ms^−1^, from −5.89 ms^−1^ to 5.84 ms^−1^, and from 5.84 ms^−1^ to 21.29 ms^−1^, the slopes are determined to be 0.37%/ms^−1^, 0.17%/ms^−1^, and 1.24%/ms^−1^, respectively. The asymmetrical nonlinear response of the sensing device to the airflow rate can be attributed to three factors. Firstly, in response to variations in airflow, the cantilever beams undergo nonlinear flexural deformation^35^. In particular, the PDMS cantilever beam exhibits slight deformation in response to low-velocity airflow, resulting in a relatively weak device response. Secondly, the presence of nylon fibers on the cantilever beam results in the reflective film bending more easily downward, thereby increasing sensitivity under positive airflow relative to negative airflow. Thirdly, as the film approaches the device, there is an increased rate of photocurrent magnitude (see Supplementary Information S2), which also explains why the device exhibits higher sensitivity under positive airflow than negative airflow.

In the large airflow rate range, the sensitivity in the negative airflow direction is lower than that in the positive airflow direction. This is because the cantilever structure of PDMS film will cause the deformation in the negative direction to be smaller than in the positive direction. Moreover, the fitting data analyzed under the condition of decreasing airflow aligns well with the data of increasing airflow, showing the stability of the sensing device.

**Fig. 4:**
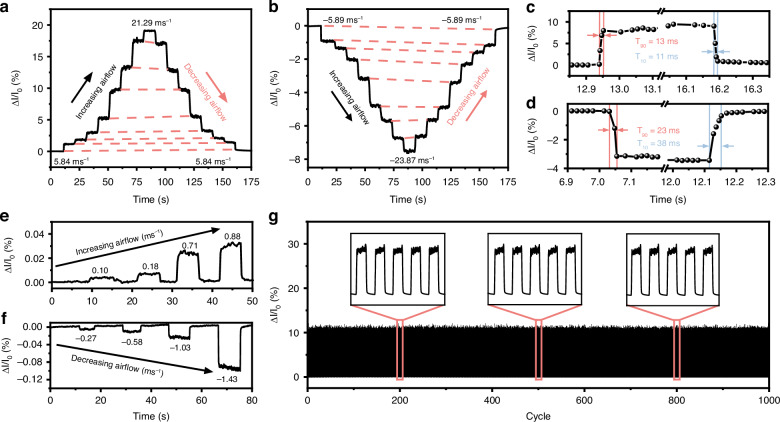
Dynamic responses of the sensing device. Photocurrent response when measuring the stepwise airflow change in **a** positive and **b** negative directions. Response time and recovery time when applying transient **c** positive and **d** negative airflow. Photocurrent response of the device under small **e** positive airflow and **f** negative airflow. **g** Reliability measurement of the device for 1000 cycles at a positive airflow velocity of 14.16 ms^−1^

The dynamic response of the sensing device is studied by obtaining the change of photocurrent with time under different airflow conditions. Figure 4a, b show the measured response by applying continuously increasing and decreasing the airflow. A highly symmetrical profile between photocurrent changes during increasing and decreasing steps, indicating consistent sensing behavior. The transient response of the device is analyzed. As shown in Fig. 4c, d, the response time *T*_90_ and recovery time *T*_10_, defined as the duration required to attain 90% and 10% of the stable level, are determined to be approximately 13 ms and 11 ms for positive airflow and 23 ms and 38 ms for negative airflow, respectively. Figure 4e, f demonstrates the response of the device to small positive and negative airflow levels, responding to airflow rates as low as 0.1 ms^−1^ and −0.27 ms^−1^, respectively. To evaluate the repeatability and stability of the device, it is subjected to 1000 consecutive cycles at a positive airflow rate of 14.16 ms^−1^. As demonstrated in Fig. 4g, the photocurrent signals exhibit high stability during the testing cycles, with highly repeatable photocurrent waveforms observed at different time periods. The stability of the device under cyclic testing at negative and low airflow velocities is detailed in the Supplementary Information S3.

Table 1 summarizes the performance comparison of the sensing device with previously reported airflow sensors. Although various advanced nanostructures and materials have been developed to enhance sensing properties, their operations are limited to a single airflow direction. The device introduced in this study offers a notable advantage with its wide measurement range spanning from −23.87 ms^−1^ to 21.29 ms^−1^, alongside achieving a remarkably low detection limit of 0.1 ms^−1^ in a compact form factor. Notably, its response time of 13 ms and 23 ms outperforms the previously reported minimum value of 40 ms^7^. This is attributed to the fast photoelectric conversion in the InGaN/GaN MQWs diode structure.

**Table 1:** Comparison of the performance of the airflow sensors

Sensing structure	Mechanism	Response time	Range	Size	Detection limit
Silicon nanowires^7^	Piezoresistive	0.04 s	0.15–15.3 ms^−1^	3 mm × 5 mm × 0.5 mm	0.15 ms^−1^
Cruciform beam/MEMS^10^	Piezoresistive	0.0241 s	−5 to 5 ms^−1^	2 mm × 2 mm × 0.3 mm	N/A
PVDF fiber^11^	Piezoelectric	1.604 s	0.5–6.5 ms^−1^	*D* = 0.3 mm, *H* = 2.75 mm	0.5 ms^−1^
Carbon fiber^18^	Piezoresistive	0.103 s	0.068–16 ms^−1^	*D* = 6 mm, *H* = 100 mm	0.068 ms^−1^
Carbon nanotubes^19^	Piezoresistive	1.3 s	0.05–7 ms^−1^	5 μm × 5 μm	0.05 ms^−1^
Carbon fiber^21^	Piezoresistive	1.7 s	0.053–2.66 ms^−1^	1 cm × 1 cm	0.053 ms^−1^
Graphene/Polyvinylidene fluoride^22^	Piezoresistive	0.55 s	3.4–10.7 ms^−1^	25 mm × 12 mm	3.4 ms^−1^
Membrane light-emitting diode^30^	Optical	1 s	1.22–2.779 ms^−1^	*D* = 1.58 mm	1.22 ms^−1^
Graphene/PDMS^8^	Piezoresistive	0.7 s	0.55–7.19 ms^−1^	7.5 mm × 5 mm × 2 mm	0.55 ms^−1^
Polymethyl Methacrylate/Polyimide^40^	Piezoresistive	0.193 s	0.5–4 ms^−1^	N/A	0.5 ms^−1^
This work	Optical	0.013 s (+ve airflow) 0.023 s (−ve airflow)	−23.87 to 21.29 ms^−1^	4 mm × 4 mm × 10.7 mm	0.1 ms^−1^ (+ve airflow) −0.27 ms^−1^ (−ve airflow)

**Fig. 5:**
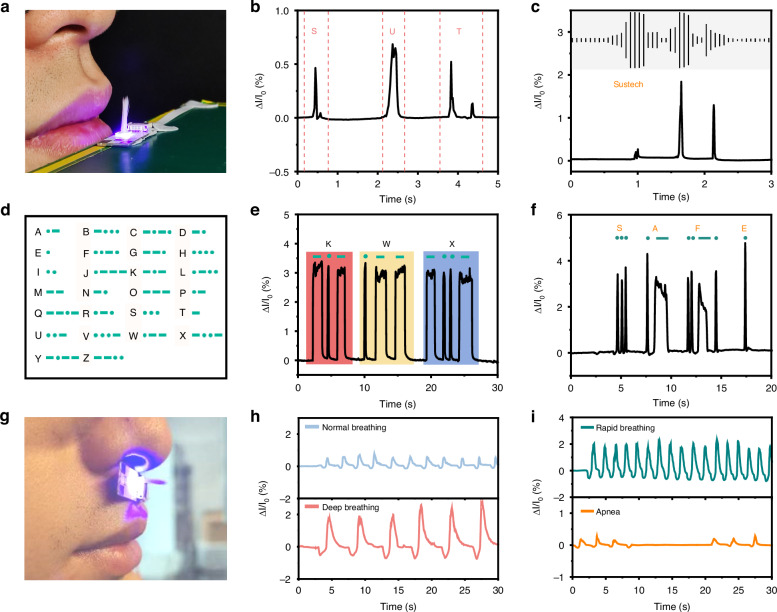
Response of the sensing device to airflow changes caused by human speech and breathing. **a** Photograph of an individual speaking in front of the sensing device. **b** Photocurrent response of the device to the airflow generated when pronouncing the letters ‘S’, ’U’, and ‘T’. **c** Photocurrent response of the device when humans pronounce ‘Sustech’. The inset shows a sound wave image of ‘Sustech’ collected with sound acquisition software. **d** Diagram showing the patterns of Morse code for different letters. Photocurrent response to airflow through nasal breathing is associated with Morse code patterns of **e** the letters ‘K’, ‘W’, and ‘X’ and **f** the word ‘SAFE’. **g** Optical image showing a person breathing through the nose into the device. Photocurrent response of the device to **h** normal breathing and deep breathing, as well as **i** rapid breathing and apnea

Having recognized the rapid response and extensive measurement range of the device, its applicability is further explored, particularly within the domain of human speech recognition. The intimate relationship between human speech and airflow, where sound is produced through modulated airflows, forms the foundation of this exploration^36,37^. As shown in Fig. 5a, the sensing device is placed in front of the human mouth, allowing for precise capture of the airflow patterns. Figure 5b shows the photocurrent variations observed when an individual pronounces the letters ‘S’, ‘U’, and ‘T’. Notably, each letter exhibits a unique waveform. Similarly, Fig. 5c shows the photocurrent variations associated with pronouncing the word ‘Sustech’. Given that ‘Sustech’ comprises three syllables, it naturally generates three separate airflow streams during the pronunciation. This characteristic is reflected in the captured sound waves, reflected in the captured sound waves segmented into three distinct parts, as seen in the figure inset. By analyzing the waveform captured by the device, it is feasible to differentiate between various spoken letters or words.

The sensing device, characterized by its rapid response and high stability, exhibits immense potential in facilitating information exchange. Specifically, patients with limb aphasia are unable to communicate with the external world through conventional means, and the utilization of breathing patterns as a method for conveying information holds significant potential for these patients. Referring to the Morse code patterns shown in Fig. 5d, each English letter can be expressed through different combinations of dots and dashes, achieved by modulating the duration of the applied airflow. For instance, Fig. 5e illustrates the encoding of the letters ‘K’, ‘W’, and ‘X’ using the airflow device. Through the encoding and decoding of information, the device can assist patients with limb aphasia in communicating with others. In simulating scenarios involving such patients, the device encodes words through nasal exhalation, as exemplified by Fig. 5f, which presents the waveform encoding the word ‘Safe’.

The versatility of the sensing device extends beyond communication assistance, as it is also employed for monitoring the human respiratory. Respiratory testing, a standard medical procedure, assesses the health of the respiratory system by measuring airflow from the mouth or nose^38,39^. The device is positioned beneath the nostril for breath detection, as shown in Fig. 5g. Figure 5h exhibits the distinct periodic waveforms in photocurrent observed during both normal and deep breathing, where exhalation and inhalation align with the positive and negative directions, respectively. Figure 5i further illustrates the capability of the device to detect rapid breathing patterns and apnea, highlighting its suitability for real-time monitoring. Notably, a distinctive feature of this device is its ability to generate two opposing peaks within each breathing cycle, representing both exhalation and inhalation, a rare characteristic among reported airflow sensors.

**Fig. 6:**
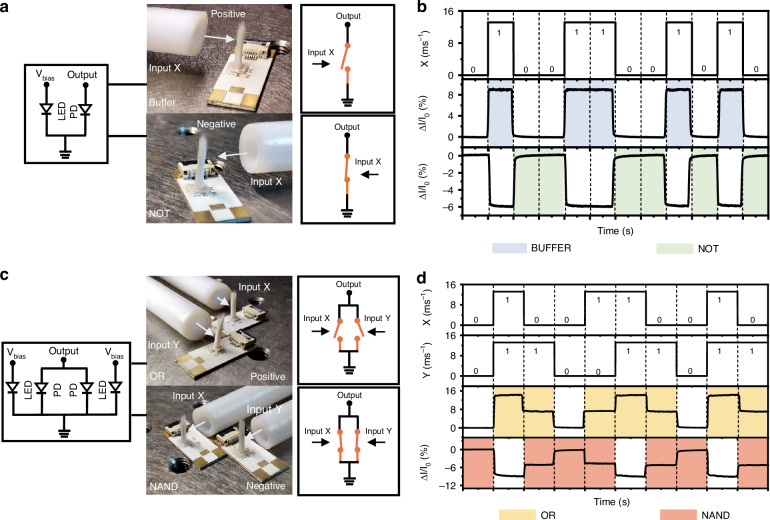
Implementation of logic operations. **a** Schematic diagrams and optical images of the device serving as a buffer and a NOT gate. The airflow applied to the device is defined as input X. **b** Photocurrent output response measured when the device works as the buffer and the NOT gate. **c** Schematic diagrams and optical images of the OR and NAND gate. The airflows applied to the two devices are defined as input X and input Y. **d** Photocurrent output response when the devices function as OR gate and NAND gate. All LEDs are driven by a constant current of 10 mA

The capability of the device to generate opposing photocurrent trends in response to positive and negative airflow reveals its potential for use in logic operations. The schematic diagram and optical image in Fig. 6a show that a single sensing device is adept at executing buffer functions. In the absence of airflow, the input is defined as a low level. After applying airflow at approximately 13.2 ms^−1^, positive airflow as a high-level input prompts an increase in photocurrent, demonstrating the behavior of a buffer. Conversely, when adjusting the device orientation to expose it to negative airflow as a high input, the device induces a noticeable decline in photocurrent. The photocurrent measured in the absence of airflow is identified as a high level, and the subsequent photocurrent reduction designates a low level. This behavior aligns with the operational logic of a NOT gate, as clearly demonstrated in Fig. 6b.

Furthermore, by connecting a pair of devices in parallel, the OR gate and NAND gate functions can be implemented, as shown in Fig. 6c. Figure 6d exhibits the recorded photocurrent output when applying various airflow input combinations. As the PDs of two devices are connected in parallel, the resultant output signal represents the cumulative sum of the photocurrent changes from both PDs. In the case of the presence of more than one airflow input, the elevated photocurrent indicates the high-level state, thus facilitating the implementation of an OR gate. Similarly, when reversing the airflow inputs to a negative direction, a NAND gate can be achieved.

## Conclusion

In summary, an optical airflow sensing device utilizing a GaN chip integrated with a biomimetic hair is demonstrated. The proposed design effectively converts the airflow dynamics into optical changes, which are subsequently captured by the GaN chip. Changes in photocurrent provide a reliable indication of airflow variations. The developed device exhibits superior performance in terms of small footprint, rapid response time, extended measurement range, low detection limit, and high stability. These advantages render the sensing device highly suitable for applications in speech recognition and information encoding. Moreover, its unique capability to detect bidirectional airflow shows great potential for breath detection and the execution of logic operations.

## Materials and methods

### Fabrication of the GaN chip

Epitaxial structures comprising InGaN/GaN multiple quantum wells (MQWs) are grown on a 4-inch c-plane sapphire substrate using metal-organic chemical vapor deposition (MOCVD). Photolithography is employed to pattern 16 hexagonal regions, each with a side length of 41 μm, as the LED, and the remaining region is defined as the PD. n-GaN surfaces are exposed using inductively coupled plasma (ICP) etching. Photolithography and ICP etching are used to completely remove the 20-μm-wide GaN region between the LED and the PD. Subsequently, p- and n-electrodes are deposited on ITO and n-GaN, respectively, through electron-beam evaporation. After the deposition of a SiO_2_ passivation layer, the distributed Bragg reflector (DBR), serving as the bottom reflector, is deposited using an optical thin-film coater. The p-pad and n-pad are deposited via electron beam evaporation. The sapphire substrate is lapped and polished, and then laser-cut into chips with dimensions of 0.9 × 1.1 × 0.16 mm^3^. The metal pads of the chip are soldered onto a printed circuit board (PCB).

### Preparation of the biomimetic hair structure

A mold with a U-shaped support cantilever beam structure is created using 3D printing. The PDMS elastomer matrix and curing agent (Dow Sylgard 184) are thoroughly mixed in a ratio of 7.5:1 wt% and then subjected to negative pressure in a vacuum pump for 30 min to remove air bubbles. A cylindrical mold prepared by 3D printing is used to control the amount and leveling of fibers bundles (See Supplementary Information S4). The mixture is poured into the mold surface and nylon fibers (8800-C1601A) with a diameter of 1.5 mm and a length of 10 mm are arranged on the top. The assembly is subsequently heated at 100 °C for 10 min. After the removal of the mold, an integrated film of nylon fiber and PDMS with a thickness of 0.2 mm is obtained. A PET reflective film (KIMT0042) with an area of 3 × 3 mm^2^ and thickness of 70 μm is attached on the bottom of the film. The nylon fibers and PDMS integrated film are affixed to the U-shaped portion of the PCB using PDMS gel, followed by a curing process to ensure adhesion.

## Supplementary information

Supplemental Material

## Data Availability

The data supporting plots within this paper and other findings of this study are available from the corresponding author upon request.
