# Supplementary material for: Biomimetic hair-assisted GaN optical devices for bidirectional airflow detection
Source: Microsyst Nanoeng. 2024 Nov 8;10:166. doi: 10.1038/s41378-024-00794-2 (PMC11544249; doi:10.1038/s41378-024-00794-2)
Supplement: Supplementary file 1 — Supplemental Material [file 41378_2024_794_MOESM1_ESM.pdf]

Supplementary Information

Biomimetic Hair-Assisted GaN Optical Devices for Bidirectional Airflow Detection

Tianyu Ye[1,#], Jian Chen[1,2,#], Xinke Tang[2], and Kwai Hei Li[1,2,*]

[1]School of Microelectronics, Southern University of Science and Technology, Shenzhen 518055, China

[2]Pengcheng Laboratory, Shenzhen 518055, China

Email addresses: Tianyu Ye (12232549@mail.sustech.edu.cn), Jian Chen (12331324@mail.sustech.edu.cn), Xinke Tang (e-mail: tangxk@pcl.ac.cn)

[#] These authors contributed equally: Tianyu Ye, Jian Chen.

[*] Author to whom correspondence should be addressed. Electronic mail:

khli@sustech.edu.cn. Tel: (+86) 075588010176. FAX: (+86) 075588010197.

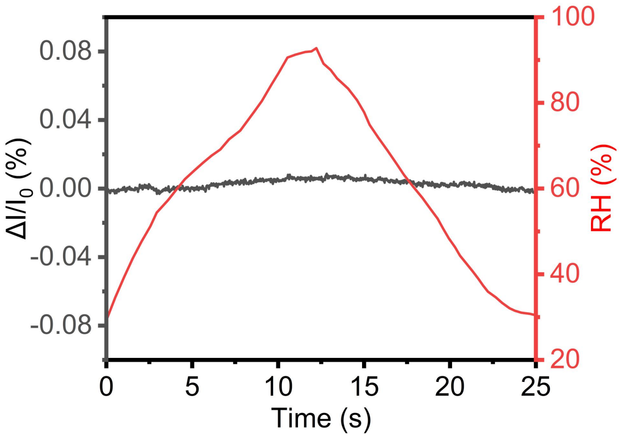

Fig. S1 Plot of the photocurrent change of the device in response to relative humidity (RH) changes of 30-90%.

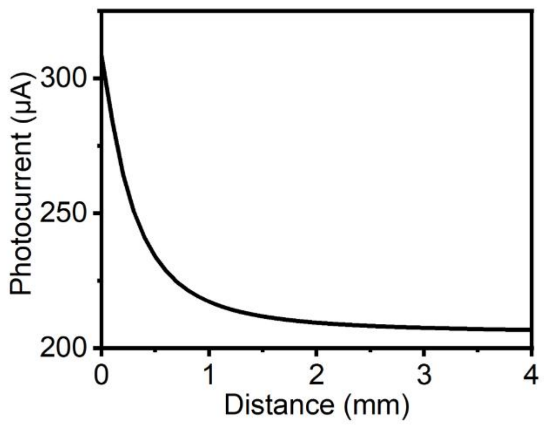

Fig. S2 Photocurrent response of the GaN chip when the PET film is positioned at different distances from the chip.

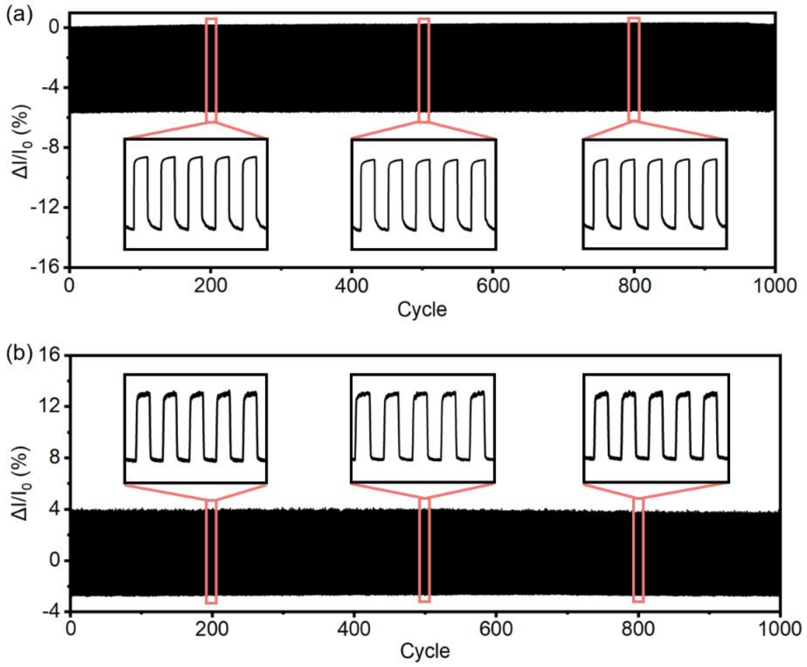

Fig. S3 Reliability measurement of the device for 1000 cycles at (a) a negative airflow velocity of 0 to -17.3 ms^{-1} and (b) a small airflow velocity of -9.0 to 8.8 ms^{-1}.

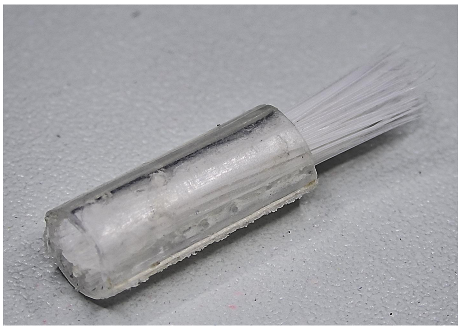

Fig. S4 Optical image of a cylindrical mold used to control the amount and of leveling of the nylon fibers.
